# Supplementary figures and images for: Following successful anti-leishmanial treatment, neutrophil counts, CD10 expression and phagocytic capacity remain reduced in visceral leishmaniasis patients co-infected with HIV
Source: PLoS Negl Trop Dis. 2022 Aug 15;16(8):e0010681. doi: 10.1371/journal.pntd.0010681 (PMC9410551; doi:10.1371/journal.pntd.0010681)

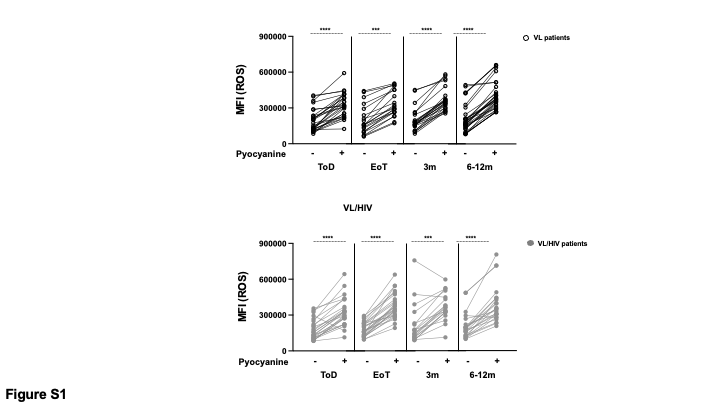

Supplement: S1 Fig — Neutrophils were isolated by dextran sedimentation from the blood of VL (ToD: n = 28, EoT: n = 20, 3m: n = 25, 6-12m: n = 33) and VL/HIV (ToD: n = 22, EoT: n = 27, 3m: n = 19, 6-12m: n = 23) patients and incubated in the absence (-) or presence (+) of pyocyanin. The production of ROS was evaluated by flowcytometry. Each symbol represents the value for one individual. Statistical differences between the MFI obtained in the absence and the presence of pyocyanin at each time point were assessed by Wilcoxon matched-pairs signed rank test. ToD = Time of Diagnosis; EoT = End of Treatment; 3m = 3 months post EoT; 6-12m = 6–12 months post EoT. ns = not significant. (TIFF) [file pntd.0010681.s001.tiff]
