# Supplementary material for: Following successful anti-leishmanial treatment, neutrophil counts, CD10 expression and phagocytic capacity remain reduced in visceral leishmaniasis patients co-infected with HIV
Source: PLoS Negl Trop Dis. 2022 Aug 15;16(8):e0010681. doi: 10.1371/journal.pntd.0010681 (PMC9410551; doi:10.1371/journal.pntd.0010681)
Supplement: Attachment — Submitted filename: Takeleetalreviewerscomments130722.docx [file pntd.0010681.s002.docx]

**Reviewers’ comments**

We thank the reviewers for their positive and constructive comments. In agreement with their comments, we have now made substantial changes, highlighted in yellow, to our manuscript (Takeleetalrevised130722). For clarity, in our response to the reviewers’ comments below (Takeleetalreviewerscommnets130722), the questions of the reviewers are highlighted in yellow, and our responses are underlined.

The manuscript is well written and the experiments well carried on. However, the referees pointed out some changes that can improve the manuscript and it will be important to observe the results and tabels as well as the statistical analysis.


Reviewer's Responses to Questions

<b>Key Review Criteria Required for Acceptance?</b></br></br>
As you describe the new analyses required for acceptance, please consider the following:</br></br>

<b>Methods</b></br></br>
-Are the objectives of the study clearly articulated with a clear testable hypothesis stated?</br>
-Is the study design appropriate to address the stated objectives?</br>
-Is the population clearly described and appropriate for the hypothesis being tested?</br>
-Is the sample size sufficient to ensure adequate power to address the hypothesis being tested?</br>
-Were correct statistical analysis used to support conclusions?</br>
-Are there concerns about ethical or regulatory requirements being met?</br></br>

Reviewer #1: (No Response)

Reviewer #2: *The results have to be expressed as means±standard deviation that measures the dispersion of individual data relative to its mean. Depending on the distribution of the values, is better to employ a median±interquartile range. Then, means±standard error of means is not applicable to the data presented in this manuscript.*

As per this reviewer’s comment, we have changed all the figures, tables, as well as the legends of all the figures and the tables of this manuscript, the data are now expressed as median with interquartile range.

*Reviewer #3: This is fine*

*--------------------

<b>Results</b></br></br>
-Does the analysis presented match the analysis plan?</br>
-Are the results clearly and completely presented?</br>
-Are the figures (Tables, Images) of sufficient quality for clarity?</br></br>

Reviewer #1: (No Response)*

*Reviewer #2: 1) The comparison with controls groups only is not adequate, once it does not consider the impact of HIV infection on the VL/HIV results :*

We have recently shown in a study performed over three years in Ethiopia that whereas patients with visceral leishmaniasis alone did not relapse after clinical cure, >78% of VL patients co-infected with HIV experienced visceral leishmaniasis relapses [1]. Therefore, the aim of our studies is to identify immunological differences between VL and VL/HIV, to help us understand the mechanisms resulting in visceral leishmaniasis relapses in VL/HIV co-infected patients. In this context, an HIV control group is unrelated. The use of a control group was important, as this group of healthy non endemic controls (HNEC) was used to show that the majority of parameters we analysed were significantly impaired in both cohorts of patients at time of diagnosis; however, the majority of these parameters were restored over time in VL patients, as they were similar to those of HNEC, this was not the case in VL/HIV co-infected patients.

*On the other hand, as the two groups of patients were follow-up until the 6–12 months after therapy, it was possible to evaluate the impact of the treatment on the neutrophil counts and functionality. Then, the comparisons between VL and VL/HIV will better evaluate the impact of HIV infection on the results presented by VL.*

All but 3 VL/HIV patients were already on ART when they were diagnosed with visceral leishmaniasis (page 7, lines 129-130) and these patients received different treatment regimens [1]. There were not enough individuals in each group to assess the impact of ART treatment on neutrophils. This has now been discussed on page 17, lines 362-370.

*2) The format of the tables has to be revised. Indeed, the titles are not adequate as they do not explain the contents of the results presented.*

In agreement with this reviewer, we have revised the titles of the tables (pages 36-49).

*The control group should be removed - see comments above.*

Please see our answer to point 1) Reviewer 2: The use of a control group was important, as this group of healthy non endemic controls (HNEC) was used to show that the majority of parameters we analysed were significantly impaired in both cohorts of patients at time of diagnosis; however, the majority of these parameters were restored over time in VL patients, as they were similar to those of HNEC, this was not the case in VL/HIV co-infected patients.

Reviewer #3: The results are clearly presented. Figure legend may be clearer (see details in general comments)

In agreement with this reviewer, we have now developed all the legends of the tables, see pages 19-23.

b>Conclusions</b></br></br>
-Are the conclusions supported by the data presented?</br>
-Are the limitations of analysis clearly described?</br>
-Do the authors discuss how these data can be helpful to advance our understanding of the topic under study?</br>
-Is public health relevance addressed?</br></br>

Reviewer #1: (No Response)

Reviewer #2: No

Reviewer #3: The conclusions are supported by the data, the detail of the mechanisms involved are not investigated but discussed in the discussion section.

--------------------

<b>Editorial and Data Presentation Modifications?</b></br><br/>
Use  this section for editorial suggestions as well as relatively minor modifications of existing data that would enhance clarity. If the only modifications needed are minor and/or editorial, you may wish to recommend “Minor Revision” or “Accept”. 

Reviewer #1: (No Response)

Reviewer #2: No applicable

Reviewer #3: The abstract would be clearer if rewritten, not starting by "As compared to..."

In agreement with the reviewer, we have amended the abstract, page 2, line 26-30.

--------------------

<b>Summary and General Comments</b></br></br>
Use  this section to provide overall comments, discuss strengths/weaknesses of the study, novelty, significance, general execution and scholarship. You may also include additional comments for the author, including concerns about dual publication, research ethics, or publication ethics. If requesting major revision, please articulate the new experiments that are needed.

Reviewer #1: This brief paper provides new information on various aspects of neutrophil biology in VL patients in comparison to patients with VL/HIV co-infection, the later representing a sizable part of the VL patient population in Ethiopia.  The impressive size of the patient cohorts studied is a major strength of these studies.   The paper is well written and the data appropriately presented.  

 While neutrophil dysfunction in VL patients, reflected by their low numbers, activation status and effector functions, were described by the same group previously, the paper extends the time of followup post-treatment to show the recovery of most of these measures.  By contrast, the neutrophils from VL/HIV patients failed to be restored in their numbers, phagocytic capacity, and expression of CD10, which is an endopeptidase whose expression is associated with activated neutrophils.     The authors conclude that the failure to restore neutrophil numbers and effector functions following treatment in VL/HIV patients suggests that neutrophils play a key role in the poor prognosis of these patients.    The problem with this argument, however, is that the failure to restore neutrophil numbers and CD10 expression were only observed in the VL/HIV patients who relapsed their VL, not the non-relapsed patients, and thus may be secondary to their VL relapse.

Whereas the neutrophil counts and the CD10 expression was higher in VL/HIV patients who did not relapse as compared to those who relapsed, these values were still significantly lower as compared to controls. Therefore, neutrophil counts and CD10 expression were not restored in VL/HIV, whether they did or did not relapse. This was unclear in our manuscript, we have now clarified these points on page 11, lines 212-215 and on pages 12-13, lines 247-250.

In tis respect, these findings are not novel, since the authors have already described neutrophil impairment in patients with primary VL.

We agree with this referee that we have previously shown that neutrophils in VL patients are activated and display impaired effector functions, we did cite our paper in the introduction of the original version of this paper (page 6, lines 107-108). This study was performed at time of diagnosis and end of treatment. In the current study we further analysed neutrophils 3 and 6-12m post the end of treatment. Furthermore, to the best of our knowledge, this is the first study evaluating neutrophil counts, activation markers and effector functions in VL/HIV co-infected patients over time.

The one parameter that was not restored in the non-relapsed VL/HIV patients was phagocytic function.

Among the parameters we assessed, we found three that were not restored in VL/HIV patients at the end of the follow-up, not *one* as stated by this reviewer: Neutrophil counts, CD10 expression and phagocytosis.

This distinction should have been more fully discussed.

We have now discussed in more detail about the impaired phagocytic abilities of neutrophils from VL/HIV patients on pages 15-16, lines 319-324 and page 17, line 367.

Reviewer #2: Previous results from the group (Yizengaw et al 2016) have already shown that neutrophils activity was altered in active VL patients. In this manuscript, the authors extended the studies to VL/HIV patients and compared them to other VL alone and healthy controls. The patients were follow-up at 4 different time points from the active VL up to 6-12 months after the end of anti-leishmanial therapy. The majority of VL/HIV patients were under antiretroviral therapy. Neutrophil studies were based on cell counts in blood, expression of molecules related to activation (CD63 and CD62L), neutrophil segmentation (CD10) and neutrophil function (arginase, ROS, phagocytosis of particles). In comparison to VL alone patients, VL-HIV patients did not recover neutrophils counts and maintained reduced phagocytic ability at 6-12 after therapy. The subject is interesting and the study was well conducted, however, as presented here the results are not suitable to be published.
Major comments:
1) The results related to the inability of VL/HIV patients to recover neutrophils counts VL are interesting but are predictable due to the impaired bone marrow haematopoiesis. It can render immature neutrophils as suggested by lower expression of CD10.

We agree with the reviewer that the inability of VL/HIV patients to recover neutrophil counts was expectable; and indeed, our hypothesis was stated in the abstract as follows: “neutrophil activation status and effector functions are fully restored in VL, but not in VL/HIV patients” (page 2, lines 31-33). However, this had never been shown previously and to the best of our knowledge, this is the first study evaluating neutrophil counts, activation markers and effector functions in VL/HIV co-infected patients over time. We discussed impaired haematopoiesis in more detail, on page 15, lines 303-310 and CD10, page 17 and line 347-361**.**

2) The authors suggested that “the poor prognosis of VL/HIV patients is likely to be further worsened by the inability of neutrophils from VL/HIV patients to phagocytose bacterial particles”. However, there was no statistical difference when VL/HIV relapsed and no relapsed patients were compared.

We are not sure to understand what this reviewer means. The ability of VL/HIV patients to phagocytose bioparticles was consistently low, with no significant improvement over time, as compared to VL patients and controls. Furthermore, the ability of VL/HIV patients who did or did not relapse over time to phagocytose bioparticle was impaired, and significantly lower as compared to controls. Therefore, we wrote that “the poor prognosis of VL/HIV patients is likely to be further worsened by the inability of neutrophils from VL/HIV patients to phagocytose bacterial particles, from the time of VL diagnosis throughout follow-up”. This is discussed on pages 15-16, lines 317-324.

Reviewer #3: In this manuscript the authors have compared the number, activation status and effector functions of blood neutrophils between VL/HIV and VL patients at the time of VL diagnosis and after antileishmanial treatment. They have observed that both cohorts of patients experienced neutropenia, increased activation and impaired effector functions compared to neutrophils from healthy controls. However, while antileishmanial treatment restored the parameters assessed to basal levels in VL patients, neutrophils counts and CD10 expression remained significantly lower in VL/HIV patients. This work follows an Ethiopian cohort over a year, providing interesting insights into the  importance of the persisting impaired neutrophil counts and response observed in VL/HIV patients after anti-leishmanial treatment. 

Major comments:
-       The first major finding of this study is the maintenance of low neutrophil counts in VL/HIV patients. The authors should better discuss the possible mechanisms linked with this process.

We have extended our discussion on the possible mechanisms that may result in neutropenia, on page 15, lines 300-315.
-       The second important finding is the low expression of CD10 in neutrophils of VL/HIV patients, that persist after treatment. The peptidase activity of CD10 may or not promote inflammation depending on many factors including the presence of the substrate. This should be clarified in the discussion.

We have extended our discussion on CD10, on page 17, lines 347-361.

In line with their hypothesis, have the authors assess if markers of inflammation are more elevated in the blood of VL/HIV patients compared to that of VL patients. It seems as they performed this analysis in recent publication (supplementary figure) using the same cohort. This should be included in the discussion.

We measured markers of inflammation, and these are published in our previous study [1]. Our results show that the levels of inflammation are significantly higher in VL/HIV patients as compared to VL patients. This has been included in the introduction, page 5, lines 87-89 and in the discussion, page 15, lines 312-314.
-       The role of ROS is said to be detrimental however, ROS has also been shown to destroy parasites, and be beneficial in VL, this duality should be better discussed.

We have extended our discussion on ROS, on page 16, lines 326-330.

-       The authors have introduced the role of neutrophils in general and in VL. The authors should also include the effect of HIV on neutrophils in the discussion of their results.

We have introduced the role of neutrophils in HIV infection in the discussion, page 14, lines 278-283 and page 17, lines 363-368.

-       Please comment on the inclusion of non-endemic healthy controls in the study, and the possible differences that could have been observed using endemic healthy controls.

The majority of VL/HIV and VL patients treated at the Leishmaniasis treatment and research Center in Gondar are migrant workers coming from nonendemic areas. Few studies have been performed in Ethiopia to study the prevalence of asymptomatic among this population, but no studies have investigated the impact of asymptomatic infection on neutrophils.

The use of a healthy non endemic controls (HNEC) was important as it was used to show that the majority of parameters we analysed were significantly impaired in both cohorts of patients at time of diagnosis; but that the majority of these parameters were restored over time in VL patients, as they were similar to those of HNEC, this was not the case in VL/HIV co-infected patients.

Minor comments:
-       The authors may consider not to start the abstract with “As compared”…

We have amended the abstract, page 2, lines 26-30.
-       The authors have previously shown that 78.1 % of VL/HIV patients relapse over 3 years. In the   present study, approximately 30% of VL/HIV patients relapse over 1 year. Are more relapses observed after the first year following infection?

We are unclear how this reviewer concludes from the current study that they are approximately 30% of VL/HIV patients who relapse after 12 months, we didn’t discuss these data as they were already published in our previous study [1]. This paper showed that 70% had relapsed by 12m, this number reached 78.1% after three years [1]. We specified in the Materials and Methods section of the current paper that we used the same cohort of patients (page 7, line 122-123).

-       The titles of the figures and tables should be more informative. Figure S1 does not have a title.

We have amended the titles of the figures (pages 19-22) and the tables (pages 36-49), as well as added a title to Figure S1 (page 24)

-       Please pay attention to the use of abbreviations, for instance, the term ROS is introduced three times: in the introduction, in the methods and in the discussion.

This has been amended throughout the text.

-       Some sentences are very long, separated by ; for instance p. 19 . the sentence starting with CD63, is 8 lines long.  Please shorten .

This has been amended, page 11, lines 216-220.

-       Please update the reference 24

This has been amended.

--------------------

PLOS authors have the option to publish the peer review history of their article (<a href="<https://journals.plos.org/plosntds/s/editorial-and-peer-review-process#loc-peer-review-history>" target="_blank">what does this mean?</a>). If published, this will include your full peer review and any attached files.<br><br>

If you choose “no”, your identity will remain anonymous but your review may still be made public.<br><br>

<b>Do you want your identity to be public for this peer review?</b> For information about this choice, including consent withdrawal, please see our <a href="<https://www.plos.org/privacy-policy>" target="_blank">Privacy Policy</a>.

Reviewer #1: No

Reviewer #2: Yes: Alda Maria Da-Cruz

Reviewer #3: No

1.	Takele Y, Mulaw T, Adem E, Shaw CJ, Franssen SU, Womersley R, et al. Immunological factors, but not clinical features, predict visceral leishmaniasis relapse in patients co-infected with HIV. Cell Rep Med. 2022;3(1):100487. Epub 2022/02/03. doi: 10.1016/j.xcrm.2021.100487. PubMed PMID: 35106507; PubMed Central PMCID: PMCPMC8784791.
